# Neonatal Hemodynamics: From Developmental Physiology to Comprehensive Monitoring

**DOI:** 10.3389/fped.2018.00087

**Published:** 2018-04-05

**Authors:** Sabine L. Vrancken, Arno F. van Heijst, Willem P. de Boode

**Affiliations:** Department of Perinatology (Neonatology), Amalia Children’s Hospital, Radboud Institute for Health Sciences, Radboud University Medical Center, Nijmegen, Netherlands

**Keywords:** neonate, hemodynamics, developmental physiology, cardiac output, hemodynamic monitoring, preterm infant

## Abstract

Maintenance of neonatal circulatory homeostasis is a real challenge, due to the complex physiology during postnatal transition and the inherent immaturity of the cardiovascular system and other relevant organs. It is known that abnormal cardiovascular function during the neonatal period is associated with increased risk of severe morbidity and mortality. Understanding the functional and structural characteristics of the neonatal circulation is, therefore, essential, as therapeutic hemodynamic interventions should be based on the assumed underlying (patho)physiology. The clinical assessment of systemic blood flow (SBF) by indirect parameters, such as blood pressure, capillary refill time, heart rate, urine output, and central-peripheral temperature difference is inaccurate. As blood pressure is no surrogate for SBF, information on cardiac output and systemic vascular resistance should be obtained in combination with an evaluation of end organ perfusion. Accurate and reliable hemodynamic monitoring systems are required to detect inadequate tissue perfusion and oxygenation at an early stage before this result in irreversible damage. Also, the hemodynamic response to the initiated treatment should be re-evaluated regularly as changes in cardiovascular function can occur quickly. New insights in the understanding of neonatal cardiovascular physiology are reviewed and several methods for current and future neonatal hemodynamic monitoring are discussed.

## Introduction

Every day, neonatologists continually struggle to determine hemodynamic instability in their patients (does normal blood pressure guarantee an adequate perfusion and oxygen delivery?), to decide to initiate treatment (should every patient with hypotension be treated or is permissive hypotension an option?) and if so, which therapy is the best for this individual patient (inotropes, vasopressors or fluid administration)? Many of these questions remain unanswered: most of the current treatment modalities and guidelines are based on expert opinion rather than on evidence, as clinical trials in neonates are challenging due to clinical, methodological, and ethical issues (1). Clinical assessment of hemodynamic compromise is proven to be inaccurate and adequate monitoring systems are warranted (2). The main goals of hemodynamic monitoring are (1) recognition of early (compensated) stage of shock as further progression to uncompensated shock will result in increased morbidity and mortality (3–5), (2) timely initiation of targeted therapy, (3) evaluation of the response to the initiated interventions, and (4) gain of insight in the developmental cardiovascular physiology to improve treatment strategies.

The neonatal circulation functionally distinguishes itself from the pediatric and adult circulation in many ways as the newborn population is a rather heterogeneous group with a wide range in gestational and postnatal ages, all with different levels of maturation of the cardiovascular system. Understanding of fetal and neonatal cardiovascular (patho)physiology is warranted in order to recognize hemodynamic instability in time. The developing cardiovascular system undergoes significant functional alterations in preload, contractility, afterload, diastolic filling, and intra-cardiac flow patterns compared to the circulation later in life (6). Besides knowledge of the immature function, insight in the dynamic changes during transition from fetal to neonatal life is mandatory (7). Especially in preterm infants, who are still developing their cardiovascular system, a normal fetal condition can become pathological several hours later in postnatal life. Furthermore, treatment decisions for many common clinical presentations of the (pre)term infant, such as sepsis, persistent pulmonary hypertension, asphyxia, and patent ductus arteriosus rely on applying knowledge of physiology and pathophysiology to identify optimal therapy. Adequate circulation can be defined as an adequate balance between oxygen delivery and oxygen consumption on both systemic and regional level. For an accurate circulatory assessment, monitoring of both the macro- and microcirculation is, therefore, essential.

Our objective is to review new insights in the understanding of transitional cardiovascular physiology and discuss available methods for current and future neonatal cardiovascular monitoring.

## Developmental Cardiovascular Physiology

### Oxygen Delivery and Consumption

The primary goal of blood flow is to deliver oxygen and nutrients to the cells to meet their metabolic demands and to remove carbon dioxide and waste products. There is a complex interaction between blood pressure, systemic blood flow (SBF), and systemic vascular resistance (SVR), which is regulated by the autonomic central nervous system (medulla oblongata), the peripheral nervous system, the cardiovascular system (baroreceptors and chemoreceptors), and endocrine mechanisms (8).

The amount of oxygen required by the tissue depends on the functional state of the cells. Furthermore, certain organs—the brain, heart, adrenal glands, and renal cortex—have persistently high oxygen demands, while other organs like the spleen and the skin can suffice with a lower oxygen supply. The gastrointestinal tract and skeletal muscles have variable oxygen needs. Neuro-endocrine and local vascular mechanisms regulate tissue perfusion in the compensated phase of shock. Blood flow toward vital organs (*via* high-priority vessels) is then preserved by local vasodilatation, while the vasculature of non-vital organs (*via* low priority vessels) tends to constrict in order to redistribute the blood mainly from the splanchnic vasculature. The latter might be a predisposing factor for the development of necrotic enterocolitis. During this phase, normal blood pressure is preserved. When this compensatory mechanism fails, hypotension will occur and organ blood flow further diminishes resulting in dysoxia and eventually cell death. Unless treated early, circulatory failure will result in organ failure and an increased risk of mortality and morbidity. Except for changes in oxygen levels, chemoreceptors are also senstitive to changes in blood carbon dioxide levels, resulting in local blood flow changes. Especially in critically ill ventilated (preterm) infants prevention of hyper- and hypocapnia is essential as hypercapnia can lead to cerebral hyperperfusion, while a low carbon dioxide level potentially decreases cerebral blood flow.

**Figure 1:**
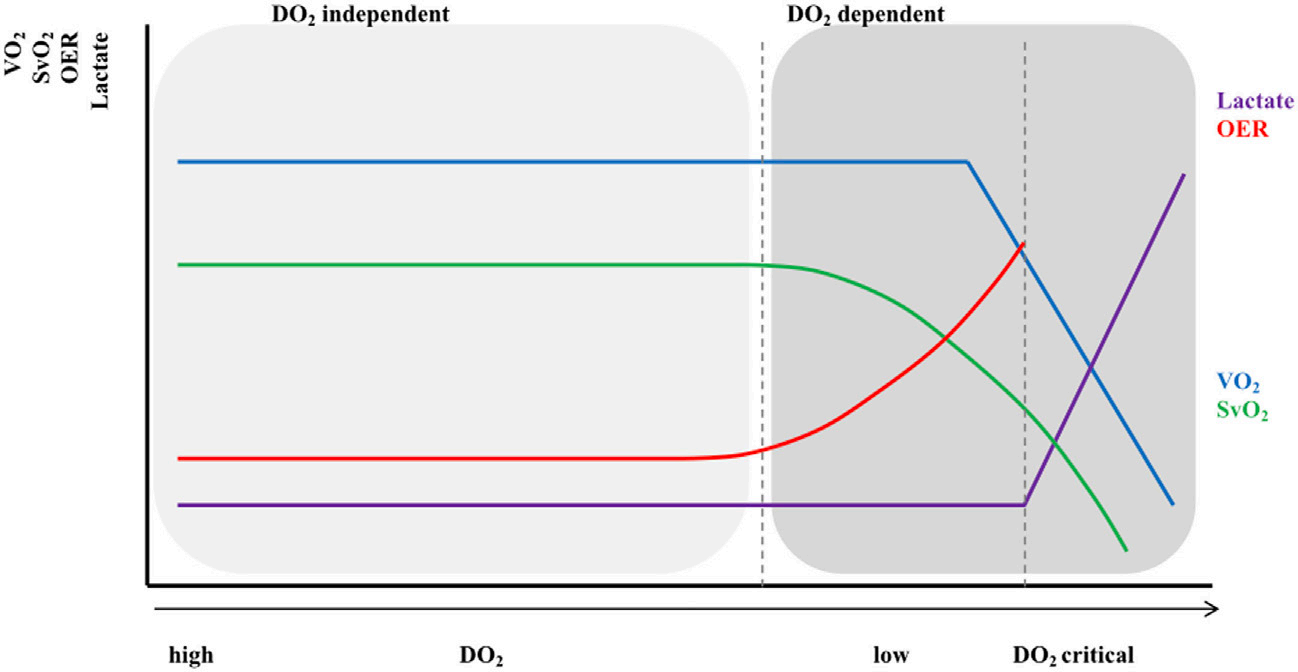
Relationship between oxygen delivery and oxygen consumption. VO_2_, oxygen consumption; DO_2_, oxygen delivery; SvO_2_, (mixed) venous oxygen saturation; OER, oxygen extraction ratio. *X*-axis shows the gradual decrease in DO_2_ with the lowest DO_2_ on the right side of the figure; Light gray area: VO_2_ = DO_2_ independent; Dark gray area: VO_2_ = DO_2_ dependent.

The relationship between tissue oxygen delivery and oxygen consumption (VO_2_) is biphasic as shown in Figure 1 (9, 10). Under normal circumstances, DO_2_ outweighs VO_2_. An increase in VO_2_ will be compensated by an increase in pulmonary oxygen uptake in patients with healthy lungs. In this circumstance, VO_2_ can be doubled without an increase in cardiac output (CO). In critically ill neonates with (respiratory and) cardiovascular compromise, a decrease in DO_2_ is compensated by an increase in oxygen extraction enabling the preservation of the level of VO_2_ (phase 1). The increase in oxygen extraction is believed to be the result of recruitment of the capillaries, thereby increasing the surface for passive oxygen diffusion. In this way, the oxygen extraction can be augmented from ±30% under normal circumstances to 50–60% when oxygen delivery is limited. These compensatory mechanisms are very important in critically ill newborn infants. However, it should be noted that it has been shown in animal studies as well as in growth-restricted fetuses and preterm infants that the high-priority vascular assignment of the forebrain is incomplete at birth and, therefore, responds with vasoconstriction when perfusion is decreased (11–14). At some critical point, this oxygen extraction ratio—defined as VO_2_/DO_2_—is maximal and a further decrease in oxygen delivery will result in a decrease in oxygen availability and, therefore, consumption oxygen (oxygen delivery-dependency) (phase 2), leading to anaerobic metabolism. The threshold for anaerobic metabolism probably occurs at SmVO_2_ <40%.

### Feto-Neonatal Cardiovascular Transition

**Figure 2:**
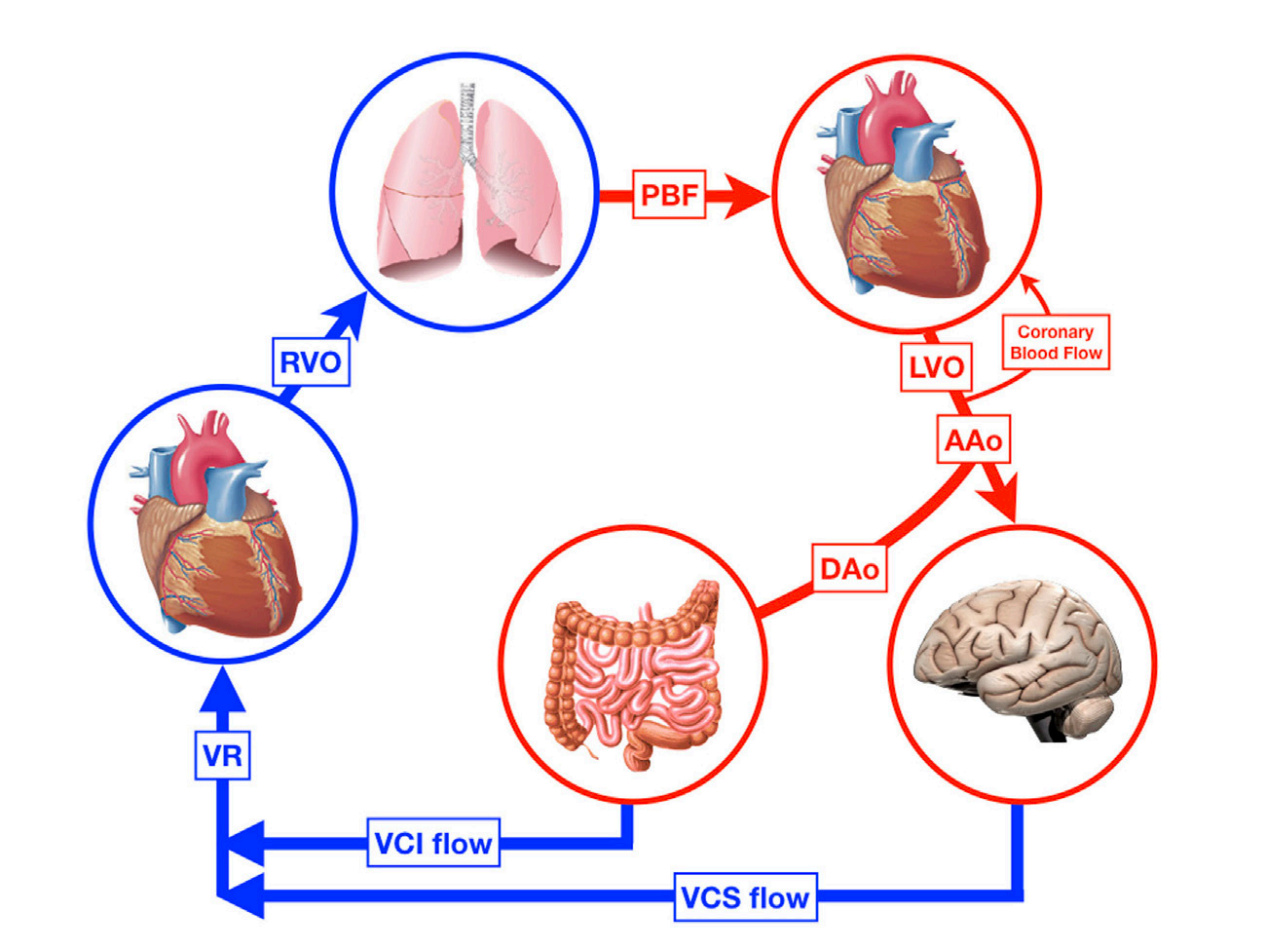
The neonatal circulation. RVO, right ventricular output; PBF pulmonary blood flow; LVO, left ventricular output; AAo, blood flow in ascending aorta; DA_o_, blood flow in descending aorta; DA, ductus arteriosus; VCS, vena cava superior; VCI, vena cava inferior; VR, venous return.

During fetal life the circulation can be characterized by two parallel circuits with equal left and right ventricle pressures, right ventricle predominance around 55% of combined CO, a low-resistance placental circulation, and the presence of shunts. Fetal animal and human studies have shown that—depending on gestational age—almost half of the total CO flows into the placenta resulting in the same amount of venous return (VR) from this low-resistance system. The umbilical venous blood passes through the ductus venosus after it has mixed with a small amount of blood from the portal vein. Most of the oxygenated blood in the right atrium is then diverted through the foramen ovale (FO) to the direction of the left atrium and left ventricle, where it is being ejected into the ascending aorta supplying the head and neck vessels together with pulmonary venous blood. The deoxygenated blood from the superior and inferior vena cava (IVC) is preferentially directed to the right ventricle, from which 75–90% of the output is then shunted through the ductus arteriosus into the descending aorta toward the rest of the body due to the higher pulmonary vascular resistance (PVR) in relation to a relatively low SVR (15, 16). Only a small portion of blood volume perfuses the lungs. With advancing gestation, changes in umbilical flow, placental perfusion, PVR, and hence pulmonary blood flow (PBF) occur, leading to a highest level of PBF around 30 weeks of gestation (17). Umbilical cord clamping at delivery is followed by major cardiovascular changes within seconds and hours, although the final transition only finalizes within weeks. The instant loss of the low-resistance, high-flow placental bed results in an increase in SVR, and a 30–50% decrease in VR affecting CO. With the onset of ventilation, PVR decreases and PBF increases followed by a rise in pulmonary VR and left atrial pressure resulting in the functional closure of the FO and completing the transition from a parallel to a serial circulation. Animal and neonatal studies have shown that after an elective caesarian section, transductal blood flow changes from predominantly right-to-left to predominantly left-to-right within 10 min after birth, reflecting the instant changes in both systemic and PVR (18, 19). The ductus venosus remains patent for several days without any circulatory consequences (20). Further completion of the transition is finalized by closure of the ductus arteriosus, which is established by multiple mechanisms, including an increase in arterial oxygen content, decreased prostaglandin levels, expression of endothelin I and its receptors, and an increase in catecholamines (21, 22). The initial functional closure of the ductus usually occurs within a few hours after birth due to smooth muscle contraction, while the anatomical closure with remodeling of the intima and loss of smooth muscle in the media might take several weeks or even months, especially in preterm infants (23). The FO can remain patent for some years. The neonatal circulation after birth is shown in Figure 2. Intrinsic and external factors can influence the transition process resulting in sustained increased vascular pulmonary resistance with persistent right-to-left shunt. In contrast, the gradually fall in PVR in combination with a higher SVR will result in left-to-right shunting when the ductus arteriosus remains patent (PDA). The incidence of a PDA correlates inversely with gestational age and weight and might increase to >50% in preterm infants weighting 500–750 g (24). A persistent patent ductus arteriosus seems to be associated with increased morbidity possibly due to pulmonary overflow in combination with decreased SBF (3, 25, 26). After many years and many non-conclusive trials, we are still not sure whether or when to treat the preterm infant with PDA (27, 28).

### Determinants of CO and Oxygen Delivery

Maintaining an optimal balance between oxygen delivery and oxygen consumption is a challenge in the transitional period and beyond. Oxygen delivery is determined by CO and the arterial oxygen content (DO_2_ = CO × c_a_O_2_); global oxygen consumption can be calculated by multiplying the CO by the difference between arterial and mixed venous oxygen content (VO_2_ = CO × c_(a-mv)_O_2_). In turn, arterial and mixed venous oxygen content are function of arterial and mixed venous oxygen saturation, arterial and venous partial oxygen pressure, and hemoglobin concentration: oxygen content cO_2_ = (Hb [mmol/L] × 0.98 [L O_2_/mmol/L Hb] × SO_2_/100) + (0.0004 × PO_2_ [kPa]).

Cardiac output is a major determinant of oxygen delivery and is defined as the product of heart rate (HR) and stroke volume (SV). SV is determined by preload, contractility, and afterload, all parameters which are intertwined. During the transitional phase, substantial changes occur in preload, myocardial contractility, and pulmonary/SVR and flow. Especially in preterm infants with an immature cardiovascular system, adaptation to these changes can be difficult.

#### SV and HR

For a long time, it was assumed that neonates can only increase their CO by increasing HR, while SV remains relatively fixed. However, several studies have shown that in term neonates during and after transition left ventricular SV and output (LVO) increases, whereas blood pressure and HR remains stable or decreases. These changes are presumably due to an increase in LV preload as a result of higher PBF in combination with ductal left-to-right shunting (29–31). Also, in term newborns with hypovolemic or cardiogenic shock, changes in LVO after volume replacement are mainly determined by changes in SV and not HR (32). Several studies indicate that changes in SBF in preterm infants are mainly the result of changes in SV rather than alterations in HR (4, 33, 34). With novel echocardiographic techniques, such as speckle tracking imaging, these findings have been confirmed (35). Furthermore, the preterm circulation is characterized by an intrinsic diastolic dysfunction and high resting HR levels resulting in a limited ability to increase CO by just increasing HR. Moreover, a rise in HR will shorten the end-diastolic ventricular filling time, decreasing SV and, therefore, attenuates CO. Most of the inotropic agents used for the treatment of circulatory failure also have chronotropic properties, partially counteracting their intended effect. In adult intensive care, trials with β-blockade (e.g., esmolol) have been shown to be beneficial in reducing HR, enhancing cardiac function, and reducing the need for vasopressors in septic patients after the initial phase of hypovolemia (36, 37). In analogy with adults, it might also be favorable to reduce HRs in tachycardic newborns to optimize SV and in addition CO. This treatment option needs further research.

#### Contractility

Contractility refers to the ability of myocytes to change their contractile force independent of preload. Due to myocardial immaturity, both morphologically and functionally, the fetus and neonate have a limited capacity to increase contractility (38). The immature myocardium is characterized by a lower number of myofibrils per cross-sectional area (CSA) with a simplified internal organization, immaturity of the sarcoplasmatic reticulum and T tubules, altered calcium handling, lower troponin C levels, and higher troponin T levels, all leading to less force during contraction (39–41). Furthermore, contractility is impaired as a result of a decreased number of β-adrenergic receptors and less sympathic innervations. The increase in contractile force (secondary to an increase in calcium influx) is age dependent and improves during the early postnatal period. Schiffmann studied the myocardial function in the developing rabbit heart and demonstrated a significant increase in contractility (45%) and relaxation (75%) within the first 8 postnatal days. Administration of increasing dosages of inotropes, such as isoproterenol and ouabain, had (significantly) lesser effect on both myocardial contractility and relaxation in neonatal hearts in comparison to adult hearts (42). The muscle fibers of the adult myocardium are organized in such a way that the squeeze of blood into the outflow tract is optimized: the apex rotates in a counter-clockwise direction to the base, while the other fibers move in the opposite direction to enhance the torsion/outflow. In preterm infants <29 weeks, James et al. found that apical rotation (counter-clockwise) remained constant over the first week, while basal rotation changed from counter-clockwise to clockwise using two-dimensional speckle-tracking echocardiography, suggesting changes in LV rotational physiology (43). Impaired myocardial function is often seen in neonates with asphyxia, preterm infants after ductus ligation, and newborns with (congenital) cardiomyopathy.

#### Preload

**Figure 3:**
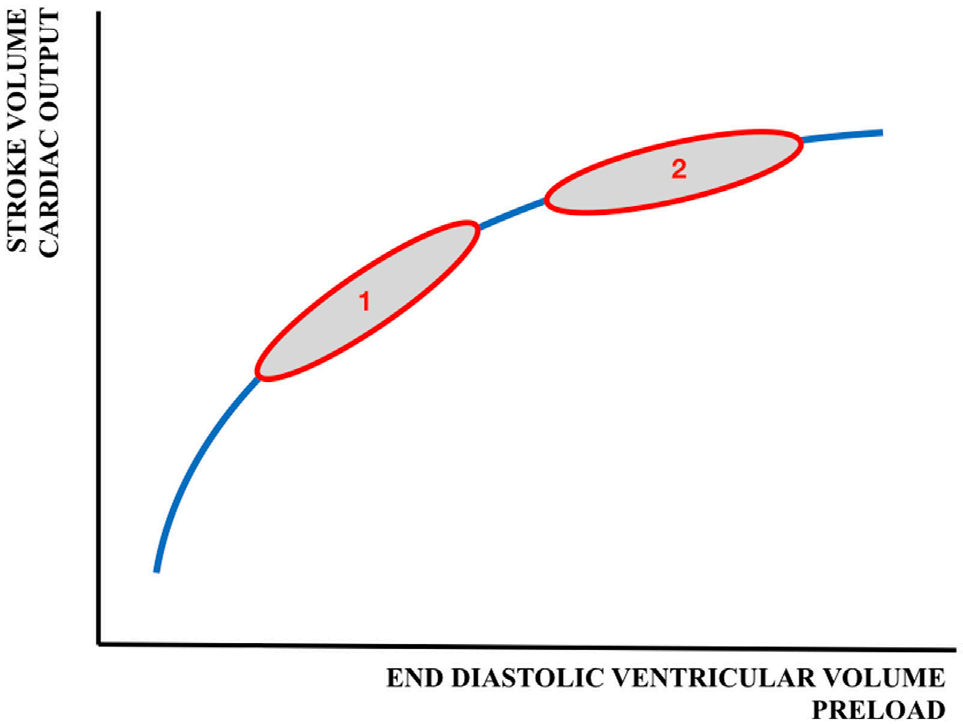
Schematic representation of the Frank–Starling curve. 1 = adult cardiac function; 2 = fetal/neonatal cardiac function. A similar change in preload will result in a larger increase in cardiac output/stroke volume in adults than in neonates.

The relationship between preload and SV is shown by the Frank–Starling (FS) curve (Figure 3). Preload refers to the ability of the heart to change its force of contraction—and, therefore, SV—in response to changes in the volume of blood entering the heart when all other factors remain constant. It is the initial stretching of the cardiac muscle fibers prior to contraction (mostly referred to as end-diastolic ventricular volume). Preload is determined by VR (and, therefore, the circulating volume and the venous capacitance) and ventricular compliance.

##### Compliance

The compliance describes the change in volume as a result of a change in pressure and depends on the structural properties of the cardiac muscle. The alteration in cardiac compliance from fetal to adult life is not yet fully understood. Early studies suggested that fetal cardiac compliance is rather low with a developmental increase in compliance during gestation (39). In contrast, more recent studies have shown that the fetal heart has a higher compliance compared to the adult heart due to the presence of specific fetal connectin isoforms (6, 44). Connectin is a protein that links the *z*-disk of the tin filament to the myosin thick filament. The fetal isoform has high elastic properties, leading to a reduced myocardial stiffness and a low passive tension. These characteristics allow the fetal myocardium to generate adequate CO despite low filling pressures *in utero*.

##### Ventricular Filling and VR

Ventricular filling is characterized by an early “passive” phase followed by an active phase, secondary to atrial contraction (E-wave and A-wave, respectively on Doppler echocardiography). The passive phase predominates in adults, children, and healty term newborns leading to an E/A-ratio >1. In contrast, in fetal diastole the active phase is more pronounced leading to an inverse E/A-ratio of <1 (45). This less passive filling is influenced by multiple factors, e.g., (1) low pulmonary VR to the left atrium due to limited PBF, so LV filling volumes mainly depend on blood flow through the FO and (2) the altered compliance: previously, the impaired passive filling was attributed to the lower compliance with increased myocardial stiffness. However, as already mentioned above, it can be argued that also the high compliant fetal heart might have a lower passive filling phase: the force generated by the connectin protein when restoring its so-called resting length after systolic contraction or diastolic distention will decrease with higher compliance (46). At early diastole, when blood is effectively suctioned into the ventricles as a result of negative pressure generated by sarcomeres restoring their resting length after systolic contraction (the ventricular untwist), the fetal myocardium will generate less force leading to less passive filling and, therefore, a lower E/A ratio.

Throughout gestation the E/A-ratio over the mitral valve will increase (but rarely exceeds >1) as the pulmonary VR (PBF) will increase (peak around 30 weeks of gestation), a process that continues after birth. Immediate cord clamping after birth leads to an increase in SVR and a decrease in VR resulting in a low LVO. LV preload will then be restored as a result of an increase of PBF in combination with a decrease in pericardial pressure when negative intrapleural pressure and lung expansion occurs, leading to a less restrictive ventricular filling pattern (47). The initial drop in LVO can partly be avoided if ventilation already commences before the cord is clamped, since then there will be no loss in preload as VR immediately switches from umbilical to pulmonary VR upon cord clamping. Initiating ventilation before cord clamping can, therefore, mitigate the changes in CO (7). In addition, delayed cord clamping is associated with other benefits, such as increased circulating volume, decreased incidence of late-onset sepsis and intracranial hemorrhage, and a reduced need for transfusions (48).

##### Circulating Blood Volume

Acute blood loss due to perinatal events (feto-maternal transfusion, placental abruption), extensive insensible water loss or capillary leak in patients with sepsis, and necrotizing enterocolitis are potential causes of low circulating blood volume resulting in a reduced preload and subsequently ventricular dysfunction. In contrast, (abundant) fluid administration might also impair ventricular function and CO as the heart functions near or on the flat part of the FS curve (Figure 3).

##### Venous Capacitance and Mean Systemic Filling Pressure (Pmsf)

The veins contain about 65% of the total blood volume. They are much more compliant than arteries, especially the splanchnic and cutaneous venous beds which have high concentrations of α1- and α2-adrenergic receptors and, therefore, sensitive to adrenergic stimulation. The venous system serves as a blood reservoir adjustable to blood flow requirements. As blood flows from the aorta into the systemic circulation, and subsequently returns *via* the veins, intravascular pressure will drop gradually to the level of the right atrium pressure (RAP). The Pmsf is defined as the equilibrating pressure across the circulatory system during circulatory arrest. Under normal conditions there is one point (the pivot point) where the intravascular pressure equals the Pmsf. The Pmsf provides, therefore, a quantitative measurement of the filling status.

**Figure 4:**
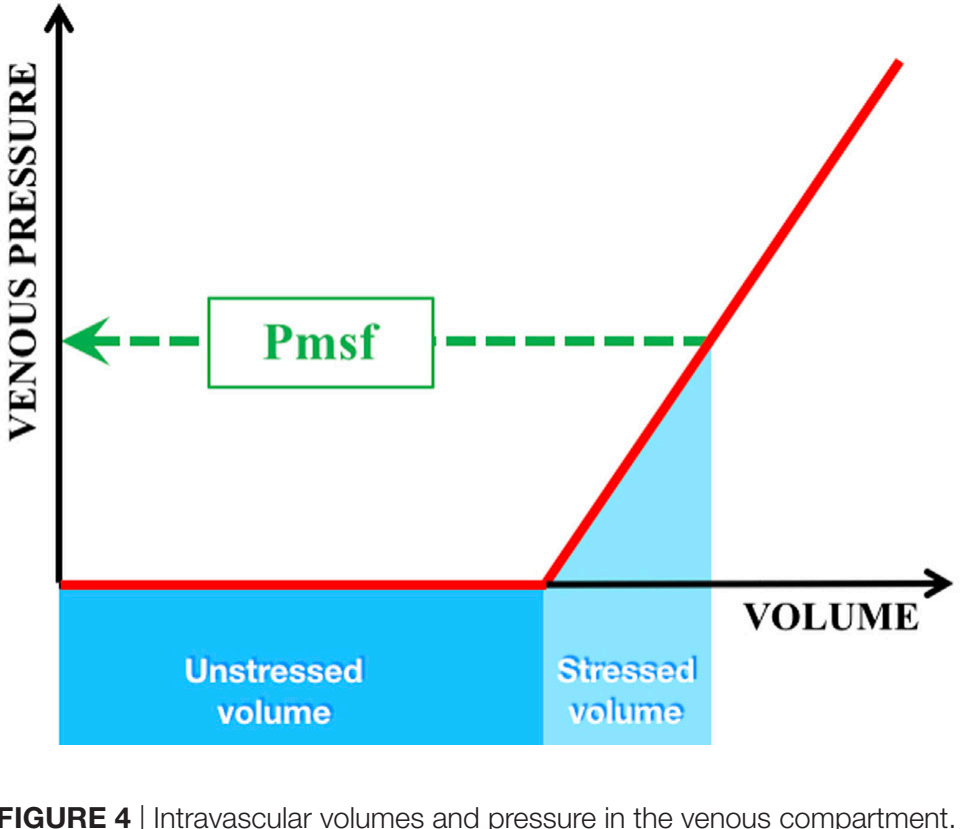
Intravascular volumes and pressure in the venous compartment. Green interrupted line = Pmsf mean systemic filling pressure; dark blue box = unstressed volume = that volume which is required to fill the vasculature without increase in transmural pressure; light blue box = stressed volume = any amount of volume added to the unstressed volume that leads to an increase in transmural pressure.

The volume that is required to fill the vasculature without increasing the transmural pressure is called the unstressed volume (Figure 4). The stressed volume (VS) is any amount of volume that leads to an increase in transmural pressure, when added to the unstressed volume. Mathematically Pmsf is calculated as the VS divided by the compliance (C) of the venous system [Pmsf = VS/C]. As the driving force for blood flow is supplied by a pressure gradient and determined by the resistance to flow (Hagen–Poiseuille’s law), the VR—which must equal CO—can be calculated by the difference between the Pmsf and RAP divided by the resistance (R_ven_) to VR [VR = (Pmsf − RAP)/R_ven_]. VR is influenced by total blood volume, compliance of the venous system, and the administration of inotropes/vasopressors (49). In addition, changes in VR will reflect on ventricular function, since CO showed equal cardiac input. Reports on the use and measurement of Pmsf are scarce in neonates (50).

#### Afterload

Afterload is defined as the force against which the heart must act in order to pump the SV and largely depends on ventricular dimensions, blood pressure, (systemic) vascular resistance, and vascular compliance. Morphologically, it is the tension experienced by each sarcomere unit. The terms afterload and ventricular wall stress are related, however, not interchangeable, as can be understood from Lamé’s equation.

$$\sigma \propto \frac{\delta P\;\times \;r}{h}=\;\frac{T}{h}$$

(σ = ventricular wall stress, δ*P* = ventricular transmural pressure, *r* = ventricular radius, *h* = ventricular wall thickness, and *T* = ventricular wall tension). With increasing afterload, the ventricular wall stress increases and the echocardiographically derived velocity of circumferential fiber shortening decreases resulting in a decrease in SV. The rate-corrected mean velocity of circumferential fiber shortening (mVcfc)—end-systolic wall stress (ESS) relation is used to measure ventricular performance, adjusting for the influence of afterload, but independent of preload and HR. Echocardiographic studies have shown that there is an age-dependent mVcfc-ESS relationship suggesting that newborns have a higher basal contractile state and that myocardial performance is more sensitive to afterload in the immature heart (51, 52). With preload—afterload interdependence, the negative effect on SV caused by an increase in afterload can partly be compensated by an increase in left atrial pressure, enhancing contractility. High afterload can be observed in neonates shortly after transition, after ductal ligation, and in the presence of cold shock (low CO and high SVR). Low afterload as a result of low vascular tone/SVR is a cause of circulatory failure in neonates with warm shock (high CO and low SVR). It is important to differentiate between these different presentations of shock, as they require other therapeutic approaches (inotropes versus vasopressors) (53–56).

#### Central Venous Oxygen Saturation

Mixed venous oxygen saturation (SmvO_2_) represents the oxygen reserve after tissue oxygen extraction. Under normal conditions the body extracts approximately 25–30% of oxygen from the arterial blood resulting in a SmvO_2_ of 70–75%. Assuming stable hemoglobin and SaO_2_ levels, a decrease in SmvO_2_ is either the result of an increase in oxygen consumption (stress, pain, seizures, or increased metabolic demand in case of sepsis), or a decrease in CO. Mixed venous oxygen saturation can only be measured from blood sampled from the main pulmonary artery, which is not feasible in neonates. An alternative is to sample venous blood from the right atrium or the caval veins (depending on the position of the central venous catheter)—central venous oxygen saturation (ScvO_2_). However, absolute values of SmvO_2_ and ScvO_2_ are not interchangeable for various reasons (57, 58). ScvO_2_ is among others influenced by (1) sampling site differences: under normal physiologic circumstances vena cava inferior oxygen saturation (SivcO_2_) is higher than vena cava superior oxygen saturation (SsvcO_2_) (the gut being a low oxygen extraction organ) with the latter being lower than the mixed venous oxygen saturation. During shock, as a result of redistribution phenomena, the relationship between the SsvcO_2_ and the SmvO_2_ might be reversed; (2) the presence of a (intracardiac) left-to-right shunt (which increases the SmvO_2_, but not SsvcO_2_); (3) incomplete mixing of blood: sampling blood from the superior or IVC might contribute to the higher values compared to SmvO_2_ as this latter also includes unsaturated blood from the coronary arteries; (4) redistribution of blood flow during shock: as cerebral blood flow and oxygenation is usually preserved over some period of shock, the drop in SsvcO_2_ is less marked and delayed compared to the drop in SmvO_2_. In contrast, due to redistribution and an increase in oxygen extraction in the splanchnic vascular bed, the SivcO_2_ will underestimate SmvO_2_; (5) the level of consciousness: restlessness can increase oxygen consumption and, therefore, decrease SmvO_2_, while (over)use of sedatives will have the opposite effect. Therapeutic cooling will also increase SmvO_2_; and (6) myocardial oxygen consumption can influence measurements of SmvO_2_. Despite these differences, absolute changes of SmvO_2_ and ScvO_2_ occur mostly in a parallel manner. While decreasing values of SvO_2_ mostly reflect inadequate oxygen delivery or increased consumption, normal or high values cannot be interpreted as normal tissue oxygenation. Especially in septic and circulatory compromised patients, SvO_2_ might be high due to mitochondrial dysfunction with impaired oxygen extraction.

#### Cardiopulmonary Interactions

**Figure 5:**
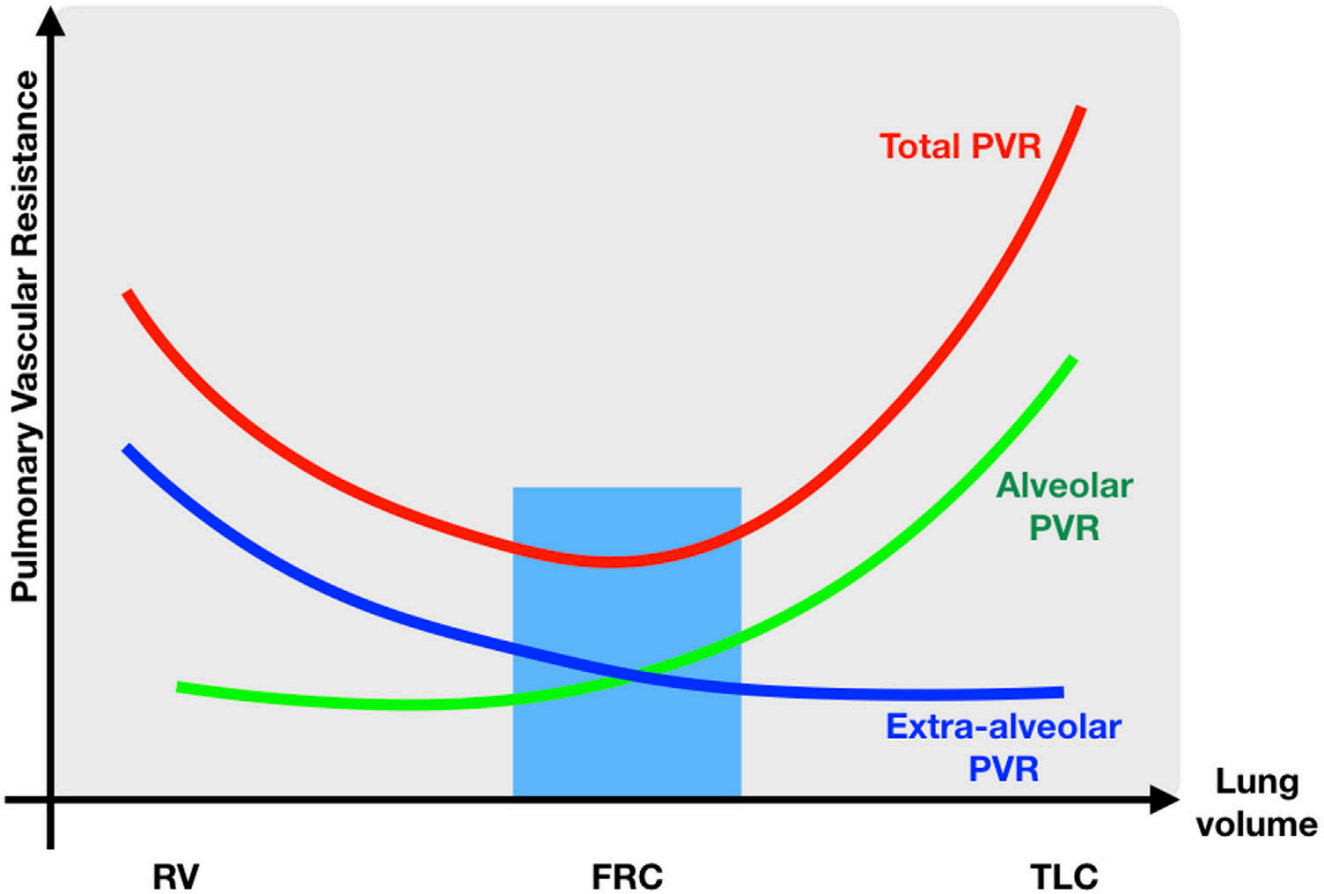
Relationship between PVR and lung volume. RV residual volume; FRC, functional residual capacity; TLC, total lung capacity; PVR, pulmonary vascular resistance; blue zone lowest pulmonary vascular resistance.

As described earlier, major changes in blood flow and blood pressure occur during normal transition. Sick newborns and especially preterm infants who have reduced respiratory drive, poor lung compliance, and reduced thoracic musculature, often require positive pressure ventilation (immediately) after birth, adversely affecting cardiovascular function in different ways (59). Increasing airway pressure decreases the alveolar/capillary transmural pressure gradient, squeezing blood out of the intra-alveolar capillaries, resulting in an increase in PVR and a decrease in PBF (Figure 5). As the LV function is highly dependent on preload, a reduction in pulmonary VR will reduce LV output. Persistent increases in airway pressure and subsequently PVR may even potentiate the persistence of a possible right-to-left shunting through the ductus arteriosus. Furthermore, a direct compressive effect of the positive airway pressure on the heart hampers the LV function and reduces CO. Inadequate ventilation strategies might not only impair cardiovascular function and SBF, but also influence cerebral blood flow in several ways, especially in preterm infants with impaired cerebral autoregulation: high positive end expiratory pressures can cause a significant reduction in CO and similarly in superior vena cava blood flow (representing also blood returning from the brain); ventilation with high tidal volumes shortly after birth results in large fluctuations in cerebral blood flow and increased vascular extravasation (60, 61). These changes in cerebral blood flow and oxygenation are associated with an increased risk of intraventricular hemorrhage and long-term neurodevelopmental disability or death (62, 63). As caution is warranted when using high airway pressures, ventilating newborns can also have beneficial hemodynamic effects, such as a reduction in LV afterload secondary to higher intrathoracic pressure, enhancing CO and oxygen delivery to the tissues. Moreover, decreasing work of breathing results in a lower metabolic demand and thereby increasing SmvO_2_.

## Hemodynamic Monitoring Systems

Continuous assessment of blood pressure, HR, and arterial oxygen saturation alone will not provide sufficient information to initiate or adapt cardiovascular interventions. Hypotension can indeed reflect high or low SBF depending on the level of SVR and oxygen delivery might be impaired despite normal blood pressure in the phase of compensated shock. Moreover, in the preterm infant, there is a tendency to accept lower blood pressure (permissive hypotension) in the absence of other signs of circulatory failure (64). The choice of treatment in the presence of hypotension can only be determined when additional information is gathered on cardiac function, as vasopressors and inotropes influence SBF and blood pressure in different ways. Simultaneously, bedside measurements of blood pressure, blood flow, and hence SVR in combination with end organ perfusion are, therefore, required to adequately monitor changes in cardiovascular function before and after therapeutic interventions. An overview of different methods of hemodynamic monitoring systems in neonatal care is shown in Table 1.

**Table 1:** Advanced hemodynamic monitoring in neonates.

Assessment of CO and intravascular volume				

Method	Hemodynamic variables	Limitations	Invasiveness and monitoring frequency	Applicability
Neonatologist performed echocardiography (NPE)	CO, vena cava superior flow, shunts, structural and functional abnormalities Left ventricular end-diastolic volume Vena cava collapsibility	Intensive training Intra-/interobserver variability 20% Error in assessment of VTI (angle of insonation) and CSA	Non-invasive Intermittent	Clinical use (absolute values of CO) Preload assessment limited for clinical use
Transcutaneous doppler (USCOM^®^)	CO	Large interobserver variability Error in assessment of VTI (angle of insonation) and CSA No anatomical verification of sample area Low precision	Non-invasive Intermittent	Limited clinical use (trend monitoring)
Thoracic electrical bio-impedance (ICON^®^, NICOM^®^)	CO	Influenced by position of surface electrodes, changes in tissue water content (pulmonary edema, pleural effusion), alterations in heart rate and motion artifacts	Non-invasive Continuous	Clinical use (trend monitoring)
Arterial pulse contour analysis (APCA)	CO	Influenced by changes in vascular compliance, vasomotor tone, medication, irregular heart rate, and motion artifacts	Invasive	Research setting
	PPV, SVV, HRV	Influenced by physiological aliasing Frequent calibration necessary	Continuous	Research setting
TPTD	CO Hemodynamic volumes (GEDV, ITV)	Use of ice-cold saline Thermistor-tipped catheter needed Arterial (femoral) and central venous catheter needed Fluid overload after multiple injections	Invasive Continuous	Only >3 kg Research setting
TPUD	CO, shunt detection and quantification Continuous COHemodynamic volumes (TEDV, CBV, ACV)EVLW	Arterial and central venous catheter needed Risk of fluid overload after multiple injections	Invasive Intermittent Continuous measurement possible (APCA)	Clinical use (absolute values of CO)APCA as trend monitoring Research setting
Stop flow method	Mean systemic filling pressure	Venous and arterial access in the same extremity Influenced by physiological factors (higher thoracic and arterial compliance and low tidal volumes compared to adults) and physiological aliasing	Invasive Intermittent	Research setting
Plethysmograph variability index	Perfusion index Fluid responsiveness		Non-invasive Continuous	Research setting

**Assessment of organ perfusion and oxygen delivery**				

Laser doppler flowmetry	Microcirculation (flow velocity)	Signal processing limitations Calibration problems Motion artifacts and probe pressure effects Influenced by skin temperature and vasopressors	Non-invasive Intermittent	Research setting
OPS, SDF, IDF	Microcirculation	Signal processing limitations (time-consuming) Effects of probe pressure Influenced by skin temperature, hemoglobin levels, and vasopressors	Non-invasive Intermittent	Research setting
NIRS	Regional blood flow, regional tissue oxygenation, and fractional tissue extraction	Lack of validation Considerable probe bias Different methods with different mathematical models Multiple assumptions Accuracy and precision questionable	Non-invasive Continuous	Clinical use (trend monitoring)

*CO, cardiac output; VTI, velocity-time integral; CSA, cross-sectional area; PPV, pulse pressure variation; SVV, stroke volume variation; HRV, heart rate variability; TPTD, transpulmonary thermodilution; GEDV, global end-diastolic blood volume; ITBV, intrathoracic blood volume; TPUD, transpulmonary ultrasound dilution; TEDV, total end-diastolic volume; CBV, central blood volume; ACV, active circulating volume, EVLW, extra vascular lung water; OPS, orthogonal polarization spectral; SDF, sidestream darkfield imaging; IDF, incident dark field imaging; NIRS, near infrared spectroscopy*.

### Methods for Measuring SBF (CO)

Cardiac output is one of the major determinants of oxygen delivery and should, therefore, be monitored continuously or at least on a regular basis. Preferably, neonatal CO monitor devices should fulfill the following clinically important conditions: they are expected to (1) be validated against a gold standard reference method and to be accurate and precise; (2) be easy applicable (bedside), non-invasive, practical, and inexpensive; (3) provide continuous information which is easy to interpret; and (4) be reliable during transition and in the presence of shunts (65). It might be clear that such an ideal monitoring system does not exist. In the past decades, many techniques have become available for the measurement of CO, based on different techniques like Doppler ultrasound, indicator dilution, and bio-impedance. However, many of these are not applicable in (preterm) neonates for various reasons, such as size restraints, need for dedicated catheters, and the inability to estimate CO in the presence of shunts (for example, during transition or in patients with congenital structural heart defects). Bedside continuous and non-invasive measurement methods are preferred, although they have the disadvantage of being less accurate and precise.

#### Non-Invasive CO Measurement Methods Applicable in Newborn Infants

##### Neonatologist Performed Echocardiography (NPE)

Neonatologist Performed Echocardiography is currently the most used non-invasive technique to estimate CO in neonatal intensive care. NPE provides extensive real-time hemodynamic information regarding intra- and extra-cardiac shunts, myocardial (dys)function, systemic and PBF, and volume changes (66, 67). Spectral analysis of the Doppler shift will produce time-velocity waveforms. The velocity-time integral (VTI) is known as stroke distance, which is the distance that a column of blood will travel during a defined time period. SV can then be calculated using the CSA of the vessel or outflow tract of interest (SV = VTI × CSA). When SV is multiplied by the HR CO is calculated. The major difficulty is the estimation of the vessel dimensions for the calculation of the CSA as CSA = π(D/2)^2^ with D the diameter of a vessel. A small error in measuring the diameter of the aorta will result in a large error for the estimation of SV and, therefore, CO, since the error is squared. Moreover, the incorrect assumption is made that the vessels are perfectly round in shape. Furthermore, in the presence of a patent ductus arteriosus with transductal left-to-right shunting, right ventricular output (RVO), and not LVO equals SBF as RVO reflects right ventricular input and, therefore, systemic VR. Some confounding by left-to-right shunting across the PFO with an increase in RVO and subsequent overestimation of SBF is, however, possible (66). Measurement of superior vena cava flow—which also reflects cerebral blood flow—can be used as a surrogate for SBF, although only from the upper body part (68). Current recommendations and guidelines to treat hypotension include NPE to target cardiovascular support and monitor the response of the initiated therapy (69, 70). The method is bedside available and non-invasive. Despite all these advantages, NPE has potential pitfalls: measurements are not continuous, intensive training and continuous practice is required, and even in capable hands a significant intra- and inter-observer variability has been observed (71). This inaccuracy is caused by the angle of insonation leading to errors in VTI and—as already mentioned—the estimation of the CSA. Training guidelines have been developed to ensure the optimal use of NPE (72). The clinical gold standard to measure CO in adults is the pulmonary artery catheter. As this method is not feasible in neonates, 2D-echocardiography is regarded as best practice in neonates and used for most validation studies with advanced hemodynamic monitoring devices, despite its relatively high error percentage of ±30%.

##### Transcutaneous Doppler Technique

The transcutaneous Doppler technique is used in the ultrasonic CO monitor (USCOM^®^, USCOM Ltd., Sydney, NSW, Australia)—a continuous wave Doppler device that is less expensive than conventional ultrasound. USCOM^®^ is designed for rapid, non-invasive measurement of CO using a probe located at either the sternal notch (aortic valve; LVO) or parasternal (pulmonary valve; RVO). CO is calculated from the measured blood flow velocity profile across the aortic or pulmonary valve. The monitor displays the VTI without visible 2D-information of the outflow tract. Results in neonates are conflicting: despite the good agreement found between RVO measurements by echocardiography and USCOM^®^ in preterm infants, precision was lacking (73). Other studies in (near) term newborns comparing CO estimates by USCOM^®^ and transthoracic echocardiography showed poor accuracy as well as low reliability with differences in RVO and LVO, irrespective of the presence of a PDA (74, 75). The authors speculated that these differences were caused by a systemic error in the calculation of the CSA of aortic and pulmonary values or by the turbulent flow in the pulmonary artery with erroneously high values of RVO. Although new users can be trained quickly, there remains a high inter-user variability. The limitations of the methods are mainly due to the use of normograms based on the patient’s height, weight, and age for estimation of the valve CSA. As absolute measurements of echocardiography and USCOM^®^ are not interchangeable at this time, the latter might be used in clinical practice as a trend monitor to detect CO changes.

##### Thoracic Electrical Bioimpedance

Thoracic electrical bioimpedance (TEB) is a non-invasive, easy applicable (four disposable surface electrodes), and continuous CO measurement method for newborns. TEB is based on impedance cardiographic technology and uses changes in thoracic electrical impedance caused by the cardiac cycle: the difference in measured voltage—produced by a small electrical current—caused by the change in alignment of red blood cells in the aorta during systole respective to diastole is used to calculate SV and CO. SV is calculated using algorithms estimating flow time, mean velocity index, and body mass. CO measurements by TEB might be influenced by the position of the surface electrodes, changes in tissue water content (pulmonary edema, pleural effusion), and alterations in HR or patient movement. Methods of TEB differ in which component of bioimpedance is utilized to create the impedance cardiogram, and in the interpretation of the waveform. Examples are Electrical Cardiometry (EC; ICON^®^, Ausculon^®^, Osypka Medical GmbH, Berlin, Germany) and the bioreactance method (Starlin^®^, NICOM^®^; Cheetah Medical Inc., Vancouver, WA, USA), where the first method analyzes the changes in signal amplitude and the latter the changes in phase shift of thoracic impedance during the cardiac cycle. Weisz et al. found that CO measurements using NICOM^®^ were feasible in neonates (GA 31–41weeks) with acceptable accuracy and precision compared to transthoracic echocardiography, but that LVO was systemically underestimated (76). They also found that in unstable extremely preterm infants after PDA-ligation, NICOM^®^ may be used merely as trend monitoring due to a large imprecision and an increasing inaccuracy over time compared to echocardiography (77). Based on preliminary data for CO estimates by EC in (pre)term newborns, body mass calculations for neonates have been adjusted in the current algorithm. Noori et al. found an acceptable agreement and precision between CO values measured by EC, and LVO measured by transthoracic echocardiography in healthy term neonates during transition (78). Grollmuss et al. presented similar results in 24 term neonates until 72 h after an arterial switch operation (79). Boet and colleagues showed good correlation with echocardiography for the measurements of SV and CO with EC in hemodynamically stable preterm infants, although there was a trend to overestimation at highest values (80). Torigoe et al. also found good agreement between CO measurements using electrical cardiometry and transthoracic echocardiography in patients with no or small PDA, but in patients with hemodynamically significant PDA CO measurement by EC seemed less reliable (81). The value of EC in assessing changes in CO in critically ill preterm and term infants in response to initiation of cardiovascular supportive care is not yet investigated. Since all studies compared TEB with transthoracic echocardiography for estimating CO, it must be emphasized that the latter is not the preferred gold standard reference technology. In our opinion TEB can be used as a trend monitor over time in clinical practice.

#### Invasive CO Measurement Methods

##### Indicator Dilution Techniques

The current use of invasive CO measurement methods is limited in neonatal intensive care. Indicator dilution techniques estimate CO based on changes of indicator concentration over time following an intravenous injection. Transpulmonary thermodilution (TPTD; PiCCO^®^, Pulsion Medical Systems, Feldkirchen, Germany) using ice-cold saline as an indicator is regarded as the clinical gold standard for estimating CO in children. As a dedicated thermistor-tipped arterial 3-French catheter needs to be inserted, this technology is not feasible in neonates weighing less than 3.5 kg.

Transpulmonary ultrasound dilution (TPUD; COstatus^®^, Transonic Systems Inc., Ithaca, NY, USA) might have potential for future use in (preterm) newborns (>0.6 kg). CO is calculated from a dilution curve that is obtained by the decrease in ultrasound velocity of the blood after injection of the indicator (isotonic saline heated to body temperature). The system uses an extracorporeal loop which can be connected in between any indwelling central venous and arterial catheter. The use of a non-toxic, low volume indicator (0.5–1 ml/kg) on body temperature makes the method attractive even for the smallest infants. TPUD provides information on CO, hemodynamic volumes, and the presence and magnitude of shunts (82). Validation studies in newborn lambs show good agreement and precision between CO measured by TPUD and ultrasonic flow probes (83). Measurements have shown to be reliable in animals with a left-to-right transductal shunt, respiratory distress syndrome, during shock and after fluid replacement (84–86). A possible limitation could be the risk of fluid overload after multiple injections of indicator. The method is feasible in infants and children (87–89). Clinical studies in neonates are in progress and reports in newborns/infants seem promising (90, 91).

##### Arterial Pulse Contour Analysis (APCA)

Arterial pulse contour analysis continuously measures CO by translating the arterial pressure waves into a beat-to-beat SV (92). Several APCA systems are available for adults, all with their own specific algorithms and assumptions regarding aortic impedance (resistance, compliance, and inductance) to translate the pressure wave form into SV. None of them are designed to take into account the specific vascular properties of children and infants. Reliability and accuracy (in children) is further hampered by changes in compliance and vasomotor tone, irregular HRs, movement artifacts, damped wave forms as a result of small catheters. The arterial pulse contour is also dependent on the site of registration. Pulse contour analysis can be used to detect changes in CO, especially when measurements are calibrated on a regular basis with another (semi-)invasive technology, such as the TPTD or TPUD. Studies in infants and children are conflicting, lack accuracy and are not convincing (93–95). Studies in neonates have not been published.

Stroke volume estimates (and in addition CO) can also be obtained by *photoelectric plethysmography* as a non-invasive continuous finger arterial pressure measurement method in adults. The Nexfin device^®^ (Edwards Lifesciences Corporation (BMeye), Irvine, CA, USA) has an incorporated arterial pulse contour CO monitor device that is applicable to the arterial waveform from the finger artery. At present only finger cuffs for adults and older children are available. The method seems feasible for blood pressure measurements in children, but further technical development is required for future CO measurement use in children and neonates as aforementioned (96).

### Assessment of Preload and Volume Responsiveness—Is It Possible?

Treatment of hypotension and circulatory failure in (preterm) newborns is challenging as interventions may be helpful, but also harmful. The most common first line treatment of hypotension is the administration of a fluid bolus, although this is associated with adverse cardiovascular effects or outcome when given unnecessarily (97). A fluid bolus will only result in an increase of preload and SV when the myocardium functions at the steep part of the FS curve (see also Figure 3). Fluid responsiveness is defined as an increase in CO or SV of more than 10–15% after volume expansion. Fluid overload (including pulmonary edema) without a resultant increase in CO will occur if fluid is administered when the patient is positioned on the flat part of the FS curve. Therefore, it would be desirable if we could predict fluid responsiveness in our neonatal population. Some interesting studies in adult and pediatric patients might guide us to new methods for future neonatal use.

Static variables—based on single observations in time—include HR, arterial blood pressure, *central venous pressure* (CVP), and end-diastolic volume estimates. Several studies in adults, children, and infants (>3 kg) have shown that CVP is a poor predictor of preload as it is influenced by multiple factors, such as right ventricular compliance, intra-abdominal pressure, and respiratory pressures (98). Although no neonatal studies have been published, CVP seems of limited value to predict fluid responsiveness in newborn infants.

Collapsibility of the IVC and LV end-diastolic volume measured by *transthoracic echocardiography* are often used to decide whether or not to give a fluid bolus. In pediatric studies, the latter parameter also failed to demonstrate an adequate predictive value, whereas the results for IVC variations are contradictory (99, 100).

The *TPUD* method can be used to estimate several hemodynamic volumes, such as total end-diastolic volume, but evidence on its value for preload assessment is still lacking (86).

Measuring *Pmsf* might be useful as it provides not only a quantitative measurement of the filling status, but also represents an estimate of the venous vascular resistance when simultaneously measured with CVP. Also, changes in Pmsf reflect changes in stressed circulatory volume. Pmsf—the pressure of equilibrium during a circulatory arrest—can be estimated at the bedside by different methods in ventilated adults, for example, the stop-flow method. This method estimates the arterial–venous equilibrium pressure (Pmsf) after proximal occlusion (stop-flow) of an extremity equipped with both an arterial and venous cannula. This is realized by inflating a cuff to a pressure above the systolic blood pressure for 30 s (101). Despite its invasiveness—requirement of a peripheral arterial catheter and preferably a peripheral intravenous access in the same extremity to measure venous pressure—the stop-flow method might be applicable in (ventilated) newborns. However, the usefulness of measuring Pmsf to predict fluid responsiveness and guide hemodynamic therapy might be limited due to its static nature and has still to be proven.

Dynamic parameters as described below are based on the variation in preload and SV secondary to cardiopulmonary interactions, for example, during positive pressure ventilation. Cyclic changes in SV between inspiration and expiration are largest when the patients’ functions on the steep part of the FS curve. This variation is also observed downstream as variable aortic blood flow, arterial blood pressure, or plethysmographic waveform amplitude. Different dynamic variables derived from *arterial blood pressure variations* have a better predictive value in adults than in children (98). The method is not yet validated in ventilated neonates, but its use is questionable as it is influenced by physiological factors (higher thoracic and arterial compliance and low tidal volumes compared to adults) and low HR/respiratory rate ratio’s (physiological aliasing), hampering the use of arterial blood pressure variations for the prediction of fluid responsiveness in newborn infants (102). The non-invasive plethysmograph variability index *PVI* is obtained from the pulse oximeter and reflects the dynamic change in perfusion index (PI) that occurs during at least one respiratory cycle (103). PI is defined as the percentage ratio between the pulsatile and nonpulsatile infrared signal. The pulsatile signal is mainly determined by arterial blood flow and the nonpulsatile signal by bone, tissue, pigments, nonpulsatile (venous and capillary) blood, and skin. Studies in children are inconclusive regarding the use of PVI to predict fluid responsiveness. Bagci et al. evaluated the technique in hypotensive neonates during surgery and suggested that PVI may predict fluid responsiveness (104). However, as measurements are subject to the same factors that influence arterial blood pressure variations, its value for predicting fluid responsiveness is limited.

### Assessment of Organ Perfusion and Oxygen Delivery

As it is not only oxygen delivery that is important for preservation of tissue integrity, but also merely the balance between oxygen delivery and consumption on both a global and regional level, additional monitoring systems are required to detect early changes in regional blood flow, and oxygen consumption and extraction. Clinical parameters in combination with additional blood gas analysis and measurement of lactate levels are mainly used to assess poor perfusion in neonates. The predictive value for circulatory failure of most of these (individual) indicators is rather limited, although changes over time or a combination of parameters may be more informative (105). As previously stated, changes in *central venous oxygen saturation* can be used as a surrogate for changes in mixed venous oxygen saturation when interpreted with caution. Although there is no linear relationship between ScvO_2_ and CO (children) and no correlation between ScvO_2_ and SBF in preterm infants (106, 107), there is some evidence that early goal-directed therapy using intermittent ScvO_2_ monitoring seems to reduce mortality rates and improve organ dysfunction in pediatric patients with septic shock (108, 109). In guidelines for the management of neonatal (catecholamine-resistant) septic shock, it is also advised to target repetitively ScvO_2_ values >70% (70). In contrast, three recent trials based on early goal-directed therapy showed that additional continuous ScvO_2_ monitoring did not improve outcome in septic adult patients who were identified early and received intravenous antibiotics and fluid resuscitation (110–112).

For the assessment of peripheral perfusion and microcirculation, a number of methods are currently available. *Laser Doppler flowmetry* uses a low-intensity laser light to measure the flow velocity of blood cells in the skin circulation. It has been shown that the neonatal microcirculation is subject to developmental changes after birth and relates to clinical illness severity scores in preterm infants. Although non-invasive and feasible in neonates, the method had some considerable (methodological) limitations (signal processing limitations, instrument calibration problems, motion artifacts, and effects of probe pressure) that prevent its clinical use at this moment (113).

*Orthogonal polarization spectral* (OPS), *sidestream darkfield* (SDF) *imaging*, and its technical successor the *incident dark field imaging* are other technologies to assess microcirculation by using light waves within the hemoglobin absorption spectrum. Quantitative measurement of the diameter of vessels, the velocity of red blood cells, and functional capillary density are made using buccal or skin measurements. OPS and SDF seem to be valuable clinical tools for the estimation of tissue perfusion in healthy and critically ill newborns (114, 115). However, temperature, hemoglobin levels, and the use of vasopressors can influence the results. Although measurements can be performed non-invasively at the bedside, results are not yet instantly available as calculations have to been performed offline and are rather time-consuming. Automated analysis will certainly facilitate future use.

*Near infrared spectroscopy* (NIRS) estimates regional blood flow, regional tissue oxygenation and—when simultaneously measured with arterial oxygen saturation—also fractional tissue oxygenation extraction, depending on the used NIRS technique. The technique uses infrared light to estimate tissue concentrations of oxy- and deoxyhemoglobin in real time. The method is non-invasive, provides continuous real-time information and is easily applicable even in the smallest preterm infants. Knowledge of regional (cerebral) blood flow and tissue oxygen extraction is useful during transition, in the phase of compensated shock with vital organ assignment (especially in the preterm, where the forebrain vasculature does not function as a high-priority structure shortly after birth as suggested by some) and during periods of hypotension with possible loss of cerebral autoregulation, all events with potential risk for adverse outcome. Although the technique is predominantly limited to clinical research, its clinical use is increasing. Observational studies in neonates evaluated the applicability of NIRS in the NICU (monitoring cerebral/splanchnic/renal autoregulation during interventions, administration of cardiovascular drugs et cetera) and beyond (intra-operatively, in the delivery room) and results are promising (116). However, when interpreting result users have to consider several limitations: (1) validation is lacking, (2) accuracy and precision are questionable, (3) probe bias is considerable, (4) different NIRS-methods use different mathematical models, signal processing, and data requirements, and (5) some assumptions have to be made (stable hemoglobin concentration, venous/arterial blood ratios, and cerebral metabolic demands are required) to use oxygen extraction as a surrogate for blood flow. At present, the large diversity and heterogenity of the NIRS studies make it difficult to draw conclusions about the clinical benefits of (cerebral) monitoring with NIRS (117, 118). Interventional studies evaluating the use of NIRS are in progress. Results of the SafeBoosC trial show that episodes of cerebral hypoxia and hyperoxia were significantly reduced in preterm infants monitored by NIRS and treated according to a dedicated guideline compared to controls, however, without any differences in short-term outcome (119).

## Conclusion

The neonatal circulation is unique and distinguishes itself from the circulation later on in life in many ways. Knowledge of the immature cardiac function during transition is essential to understand the specific pathologies and to target therapeutic interventions. Adequate and objective assessment of the systemic and peripheral circulation is, therefore, necessary, preferably in a non-invasive and continuous way. Technical advances have broadened the arsenal of monitoring systems over the past years, and although some of them might have potential for neonatal use in the near future, most of the current hemodynamic monitoring systems are not yet applicable in the vulnerable newborn. Further technical improvement is, therefore, required. It is important to recognize the limitations of the monitoring system used. And above all: monitoring itself—even if accurate or complete—will not improve outcome by itself, but rather by appropriate therapeutic interventions guided by adequate interpretation of the data obtained (120).

## Author’s Note

This review was part of the thesis of SV and is currently updated. Vrancken S. *Neonatal Hemodynamic Monitoring: Validation of an Advanced Monitoring Technique* [*Dissertation*]. Enschede: Radboud University Nijmegen. Ipskamp Printing (2016).

## Author Contributions

SV substantially contributed to the conception and design of the work, drafted the work, approved the final version to be published, and agreed to be accountable for all aspects of the work. AH substantially contributed to the conception of the work, revised it critically, approved the version to be published, and agreed to be accountable for all aspects of the work in ensuring that questions related to the accuracy or integrity of any part of the work are appropriately investigated and resolved. WB substantially contributed to the conception of the work, revised it critically, approved the version to be published, and agreed to be accountable for all aspects of the work in ensuring that questions related to the accuracy or integrity of any part of the work are appropriately investigated and resolved.

## Conflict of Interest Statement

The authors declare that the research was conducted in the absence of any commercial or financial relationships that could be construed as a potential conflict of interest.
